# Potential Ecological and Human Health Risks of Heavy Metals in Soils in Selected Copper Mining Areas—A Case Study: The Bor Area

**DOI:** 10.3390/ijerph18041516

**Published:** 2021-02-05

**Authors:** Marioara Nicoleta Filimon, Ion Valeriu Caraba, Roxana Popescu, Gabi Dumitrescu, Doina Verdes, Liliana Petculescu Ciochina, Adrian Sinitean

**Affiliations:** 1 Faculty of Chemistry, Biology, Geography, West University of Timişoara, 300115 Timişoara, Romania; marioara.filimon@e-uvt.ro (M.N.F.); adrian.sinitean@e-uvt.ro (A.S.); 2 ANAPATMOL Research Center, “Victor Babes” University of Medicine and Pharmacy, 300041 Timisoara, Romania; popescu.roxana@umft.ro (R.P.); gdumitrescu@animalsci-tm.ro (G.D.); dverdes@umft.ro (D.V.); 3 Faculty of Bioengineering of Animal Resources, Banat University of Agricultural Sciences and Veterinary Medicine “King Mihai I of Romania” from Timisoara, 300645 Timisoara, Romania; lilianapetculescuciochina@animalsci-tm.ro; 4 Faculty of Medicine, “Victor Babes” University of Medicine and Pharmacy Timisoara, 300041 Timisoara, Romania

**Keywords:** heavy metals, soils, spontaneous plants, vegetables, ecological risk, human health risk

## Abstract

Surface soil samples were collected near the Open Pit Bor (S1) and Open Pit Cerovo (S2), a grassland along the Borska Reka River (S3) and an unpolluted garden near Slatina village (reference site). Spontaneous plants (dandelion, nettle, coltsfoot, and creeping buttercup) and vegetables (onion, garlic, carrot, parsley, celery, potatoes, dill, and sorrel) were obtained from the former three sites and the reference site, respectively. The samples were analyzed for Zn, Cu, Fe, Mn, and Pb via FAAS. Pollution indices indicated low-to-moderate soil contamination at sites S1, S2, and S3. Cu was the main contaminant of environmental concern, being above the maximum admitted concentration at site S1. Metal levels in spontaneous plants were below phytotoxic levels. Cu content of leafy vegetables and celery roots and Pb content of most vegetables were not safe for human consumption. Metal concentrations tended to be significantly lower in plants than in soils, with only Cu occurring at significantly elevated levels in celery roots and sorrel leaves. Non-carcinogenic risk assessment showed that consumption of carrot roots and especially celery roots grown on unpolluted soils from the Bor area might pose long-term health risks for females and males, with the main contributors being Cu and Fe.

## 1. Introduction

It is a well-known fact that soils act as the major sink for heavy metals (hereinafter referred to as metals) that are derived from both natural and anthropogenic sources [1,2,3,4]. Given their persistence and non-biodegradability, these elements easily accumulate in soils and are then further transferred along terrestrial food chains to the upper trophic levels (primary producers, herbivores, and predators) [1,2]. Soil contamination with metals poses major risks and hazards to land ecosystems through multiple paths, with food ingestion serving as the main exposure route for humans and animals [5]. Studies monitoring metal levels in soils and assessing the risks associated with their bioaccumulation in terrestrial food webs have hence become an important research direction in this era of ever-increasing anthropogenic pressure on natural environments. Certain metals, such as copper (Cu), zinc (Zn), iron (Fe), manganese (Mn), and lead (Pb), are high-profile environmental pollutants due to their wide range of industrial applications, long history of human exploitation, and potential health risks associated with their ingestion. Cu, Zn, and Fe are among the top 10 most-commonly-used chemical elements in industrial applications [2], whereas Pb and Mn are frequently encountered as companion metals in Cu, Zn, and Fe deposits and have numerous applications in several key industries, such as the automotive industry, construction industry, or steel industry [2,6]. At physiological concentrations, Cu, Zn, Fe, and Mn function as essential elements for all living systems due their key role in metalloenzymes [7]. Beyond these ranges, however, their altered homeostasis results in metal deficiency or excess (intoxication), leading to serious adverse health effects [2,7]. By contrast, Pb serves as a non-essential element, with no known biological role in living systems and high toxicity even at trace levels of exposure [2,8].

In accordance with the 2019 Pollution and Health Metrics: Global, Regional and Country Analysis elaborated by Global Alliance on Health and Pollution (GAHP), Serbia ranks first in Europe and ninth in the world in terms of environmental pollution [9]. The city of Bor and the surrounding areas—an important mining hub—are one of the major centers of non-ferrous mining, smelting, and processing in Serbia and South East Europe [10]. With copper mining dating back to Roman times and a history of modern exploitation of copper ore spanning more than one century, this region has become a high-profile environmental hot spot in Europe [11,12,13]. Several studies already addressed the retention of metals derived from copper-mining and processing activities in soils at medium- and highly-contaminated sites from this area and reported elevated levels of Zn, Cu, Fe, Mn, and Pb [12,13,14,15]. However, there is little information regarding the levels to which these metals are found at less contaminated sites or in vegetable gardens in the Bor area. There is also little knowledge about the degree to which these elements are accumulated in native spontaneous plant species and especially in common edible plants [14]. Moreover, no study has yet investigated the non-carcinogenic health risk associated with ingestion of food crops grown in the Bor area.

In this context, this study aimed at providing environmental scientists with a multifaceted perspective on Zn, Cu, Fe, Mn, and Pb occurring in soils at a high-profile environmental hot spot in Europe by (1) determining their total levels and pollution degrees in soils impacted to different extents by anthropogenic activities; (2) evaluating metal soil-to-plant transfer in selected cultivated and non-cultivated plant species; (3) and assessing the non-carcinogenic risk related to consumption of vegetables grown in unpolluted soils from the Bor area. We used flame atomic absorption spectrometry (FAAS) to quantify Zn, Cu, Fe, Mn, and Pb concentrations in soils at four sites and in aboveground parts of four spontaneous plant species (dandelion, nettle, coltsfoot, and creeping buttercup) and edible parts of eight vegetables routinely included in the human diet (onion bulbs, garlic bulbs, carrot roots, parsley roots, celery roots, potato tubers, dill leaves, and sorrel leaves). Relevant indices, e.g., the geo-accumulation index, the enrichment factor, and the Nemerow integrated pollution index, were used to assess the soil pollution status. Statistical analysis of metal levels in plants versus soils and quantitative estimation of their potential to induce non-cancer effects via dietary consumption as per the US EPA methodology were also performed.

## 2. Material and Methods

### 2.1. Sample Localization and Collection

With about 48,000 inhabitants, the municipality of Bor lies in Eastern Serbia, 245 km away from Belgrade and approximately 100 and 30 km away, respectively, from the borders with Romania and Bulgaria. The Bor Copper Mining and Smelting Complex is located near the city of Bor and is one of the largest centers for copper mining and smelting in Europe. The copper-smelting plant together with the three mines (Open Pit Bor, Open Pit Veliki Krivelj, and Open Pit Cerovo), constitute RTB Bor, which pose serious threats to environmental health for this region and Serbia via airborne pollutants, discharged mine effluents, waste rock impoundments, and seepage from tailings [8,10].

**Figure 1:**
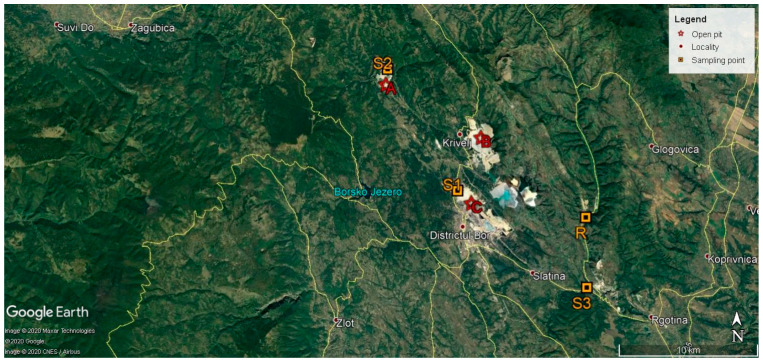
Location of the study area and sampling sites (from Google Earth). R—vegetable garden near the village of Slatina; S1—vegetation-covered mine dumps near the Open Pit Bor; S2—grassland near the Cerovo River and Open Pit Cerovo; S3—grassland near the Borska Reka River; A—Open Pit Cerovo; B—Open Pit Krivelj; and C—Open Pit Bor.

Soil samples (triplicates) were taken from the upper soil horizons (0–20 cm) at four locations, herein abbreviated as site R, site S1, site S2, and site S3. Each triplicate was obtained by randomly collecting three topsoil samples (100 g per each) from an area of 20 × 20 m (400 m^2^), mixing them together, and then sampling 50 g of soil. Site R was a vegetable garden near the village of Slatina (latitude = 44°4′34.35″ N, longitude = 22°12′35.28″ E), a low-anthropized area, which is located away from RTB Bor and other major sources of metal pollution. This location was chosen as a reference site (site R). Site S1 lay within the vegetation-covered mine dumps near the Open Pit Bor (latitude = 44°5′36.89″ N, longitude = 22°5′40.37″ E). Site S2 was a grassland near the Cerovo River, located in the proximity of the Open Pit Cerovo (latitude = 44°10′18.76″ N, longitude = 22°1′54.86″ E). Site S3 was a grassland near the Borska Reka River (latitude = 44°1′51.24″ N, longitude = 22°12′38.14″ E), situated farther from the RTB Bor than the previous two sites. The Borska Reka River (syn. the Bor River) collects the highly polluted waters of the Cerovo River, Kriveljska River, and Ravna River, and passes next to the Open Pit Bor, and is considered to be one of the most polluted water streams in Europe [8]. The latitude and longitude GPS coordinates of these sites are shown in Figure 1. The distance between our sampling points and sites A, B, and C (Figure 1) were as follows: 9270 m between site R and site B; 8240 m between site R and site C; 1320 m between site S1 and site C; 1140 m between site S2 and site C; and 10 km between site S3 and site C. The major pollution sources for the investigated sites are main dumps and tailings for sites S1 and S2 and water pollution for site S3.

Spontaneous plants were collected from sites S1, S2, and S3, whereas vegetables were obtained from the reference site (R). Sampling activities were conducted between May–October 2019. In this study, we used only plant parts with direct relevance for human nutrition and ecotoxicological risk assessment. The aerial part of plants (leaves, stems, and flowers) and the bulbs, roots, and tuberous roots were collected in triplicates, with each triplicate containing three or four specimens so that the final number of samples for each plant was 10. Each triplicate was obtained randomly from an area of 20 × 20 m (400 m^2^), within the same area as the corresponding soil samples.

In the case of spontaneous plants, i.e., dandelion (*Taraxacum officinale* F.H. Wigg, 1780; fam. *Asteraceae*), coltsfoot (*Tussilago farfara* L., 1753; fam. *Asteraceae*), nettle (*Urtica dioica* L., 1753; fam. *Urticaceae*), and creeping buttercup (*Ranunculus repens* L., 1753; fam. *Ranunculaceae*), only the aerial parts (including stems, leaves, and flowers) were sampled. With respect to cultivated plants, the following edible parts were sampled: the bulbs from onions (*Allium cepa* L., 1753; fam. *Amaryllidaceae*) and garlic (*Allium sativum* L., 1753; fam. *Amaryllidaceae*); the roots from carrots (*Daucus carota* subsp. *sativus* (Hoffm.), Arcang., 1882; fam. *Apiaceae*), parsley (*Petroselinum crispum* (Mill.) Fuss, 1866; fam. *Apiaceae*), and celery (*Apium graveolens* L., 1753; fam. *Apiaceae*); the tuberous roots from potatoes (*Solanum tuberosum* L., 1753; fam. *Solanaceae*); and the leaves from dill (*Anethum graveolens* L., 1753; fam. *Apiaceae*) and sorrel (*Rumex rugosus* Campd., 1819; fam. *Polygonaceae*).

### 2.2. Sample Preparation and Analytical Methods

The soil samples were first air dried at laboratory temperature (22 ± 2 °C), ground, sieved through a 2 mm mesh, and then sieved again through a 150 µm nylon mesh. After being oven dried at 105 °C to constant weight, they were weighed three times to the nearest 0.01 mg by using an analytical balance (model TP-214, Denver Instrument GmbH, Göttingen, Germany), with only the mean value being taken into account. Next, the samples (10 g) were ashed at 650 °C for 4 h and dissolved in 20 mL of 0.5 N HNO_3_ solution prior to being filtered through ash-free filter paper. The filtrate volume was brought to 50 mL with 30 mL of 0.5 N HNO_3_ solution.

The aerial parts were washed with double-distilled water and then oven dried to constant weight at 80 °C [16]. After being ground to pass through a 2 mm sieve, these dried samples were kept at laboratory temperature till further processing. The bulbs, roots, and tuberous roots were washed with double-distilled water and cut transversally into thin sections (1–2 mm) before being oven dried, as previously described. Next, the vegetal samples were calcinated in a muffle furnace, with the temperature being gradually increased to 650 °C and maintained constant for 4 h. The resulting ash was dissolved in 20 mL of 0.5 N HNO_3_ solution and filtered through ash-free filter paper before analysis. The volume was brought to 50 mL using 30 mL of 0.5 N HNO_3_ solution; this solution was prepared with 65% suprapure nitric acid (Merck KGaA, Darmstadt, Germany).

### 2.3. Metal Analysis and Quality Control

The concentrations of Zn, Cu, Fe, Mn, and Pb in the filtrate were assessed by using flame atomic absorption spectrophotometer with high resolution continuum source (Model ContrAA 300, Analytik Jena, Germany), fitted with specific conditions for each metal [17]. The measured values were expressed as milligram per kilogram dry weight (mg kg^−1^ dw). Mix standard solutions of trace metals analyzed (1000 mg/L), i.e., Zn, Cu, Fe, Mn, and Pb—ICP multi-element standard solution IV CertiPUR, were procured from Merck (Merck KGaA, Darmstadt, Germany). Solutions of varying concentrations were prepared for all target metals by diluting the aforementioned standards. We used only double-distilled water (spectroscopic pure) for the preparing reagents and standards. All chemicals were trace metal grade (Suprapur). Concentrate nitric acid (HNO_3_ 65%) and concentrate HCl (30%) were obtained from Merck Germany. All glassware was treated with a 20% Pierce solution (*v*/*v*), rinsed with cold tap water followed by 20% (*v*/*v*) nitric acid, and then rinsed again with double-distilled water. All blanks and duplicates samples were analyzed during the procedure to ensure the validity and reliability of the results obtained. NCS Certified Reference Material-DC 85104a and 85105a (China National Analysis Center for Iron&Steel) were analyzed for quality assurance. Percent recovery means were Zn (102%), Cu (105%), Fe (92%), Mn (95%), and Pb (94%), with all variation coefficients falling below 10%. Detection limits (μg/g), as determined by the calibration curve method, showed the following values: Zn (0.43), Cu (0.13), Fe (0.15), Mn (0.19), and Pb (0.05). The blank reagent and standard-reference soil materials were included in each sample batch to verify the accuracy and precision of the digestion procedures.

### 2.4. Pollution Assessment

To assess pollution level and associated risks we used both single and integrated hazard indices. First, we determined the index of geo-accumulation index (*I_geo_*) according to the equation:

*I_geo_* = log_2_[*C_n_*/(1.5 × *GB*)]

where *C_n_* is the concentration of target metal in the soil examined; 1.5 is a constant factor introduced to compensate possible variations in background values due to lithogenic actions; and *GB* is the value of geochemical background of target metal. The *GB* values were calculated as means of data compiled from the specialty literature (see Table 1). This index consists of seven classes: uncontaminated (*I_geo_* ≤ 0); uncontaminated to moderately contaminated (0 < *I_geo_* ≤ 1); moderately contaminated (1 < *I_geo_* ≤ 2); moderately to heavily contaminated (2 < *I_geo_* ≤ 3); heavily contaminated (3 < *I*_geo_ ≤ 4); heavily to extremely contaminated (4 < *I_geo_* ≤ 5); and extremely contaminated (*I_geo_* ≥ 5) [18].

We next calculated the enrichment factor (*EF*) using the equation given below:

*EF* = (*C_n_/C_ref_*)/(*B_n_/B_ref_*)

where *C_n_* is the concentration of target metal in the soil examined; *C_ref_* is the concentration of reference metal in the soil examined; *B_n_* is the background concentration of target metal; and *B_ref_* is the background concentration of reference metal. We used Fe as a reference metal in accordance with other studies that used this index to characterize the potential accumulation of anthropogenic metals in soils obtained from the Bor area [15,19]. Soil quality was assigned to one of the following contamination classes: deficiency to minimal enrichment (*EF* ≤ 2); moderate enrichment (2 < *EF* ≤ 5); significant enrichment (5 < *EF* ≤ 20); very high enrichment (20 < *EF* ≤ 40); and extremely high enrichment (*EF* > 40) [20].

After that, we computed the Nemerow integrated pollution index (*PI_N_*) as an integrated indicator to assess the overall degree of soil contamination based on content of all target metals:

$${PI}_{N}=\;\frac{\sqrt{{\left(\frac{1}{n}\sum_{i=1}^{n}Pi\right)}^{2}+{\left(Pimax\right)}^{2}}}{2}$$

where *P_i_* is the single pollution index of each metal, expressed as the ratio between *C_n_* and *B_n_* (terms already defined above); *P_i max_* is the maximum value of *P_i_* for all target metals; and *n* is the count of metal species. The following *PI_N_* classes were considered: no pollution (*PI_N_* ≤ 0.7); warning line of pollution (0.7 < *PI_N_* ≤ 1); low level of pollution (1 < *PI_N_* ≤ 2); moderate level of pollution (2 < *PI_N_* ≤ 3); and high level of pollution (*PI_N_* > 3).

### 2.5. Human Health Risk Assessment

Quantitative estimation of potential for non-cancer effects through consumption of metal-contaminated vegetables was conducted using the target hazard quotients (THQ) [21], as per the US EPA Region III Risk-Based Concentration Table [22]:

*THQ* = (*E_F_* × *E_D_* × *F_IR_* × *C_M_*)/(*R_f_D* × *W* × *T_A_* × 10^3^)

where *E_F_* is exposure frequency (days year^−1^); *E_D_* is the exposure duration (years); *F_IR_* is the food ingestion rate (g person^−1^ day^−1^); *C_M_* is the metal concentration in vegetables (mg kg^−1^); *R_f_D* is the oral reference dose (mg kg^−1^ day^−1^); *W* is the mean body weight for adults (kg); and *T_A_* is the average exposure time for non-carcinogens (number of days year^−1^ × number of exposure years). Exposure frequency (*E_F_*) was set to 183.5 days year^−1^ [17]. Length of exposure (*E_D_*) was considered to be 65 years for men and 70 years for women based on the mean life expectancy, starting from 8 years [23]. The values of food ingestion rate (*F_IR_*) were estimated based on data from specialty literature [17,24]. More precisely, one portion of 0.350 Kg was considered to be a daily typical serving of root vegetables or tuber vegetables (parsley, carrot, celery, garlic, and onion) and 0.150 Kg was considered to be a daily typical serving of leafy vegetables (dill and sorrel). We applied the following *R_f_D* values: Zn, 0.3 mg kg^−1^ day^−1^; Cu, 0.04 mg kg^−1^ day^−1^; Fe, 0.7 mg kg^−1^ day^−1^; Mn, 0.14 mg kg^−1^ day^−1^; and Pb, 0.0035 mg kg^−1^ day^−1^ [17,25,26]. The average body weight for adults (*W*) was set to 87.5 kg for Serbian males and 71.7 kg for females [27]. *THQ* value of 1 implies no obvious risk. *THQ* values between 1 and 4 indicate a level of concern due to exposure being above the *R_f_D*, and hence daily exposure at this level is likely to induce potentially harmful health effects throughout the lifetime in the exposed human populations. For *THQ* values above 4 there is a high level of concern, with exposed populations being highly likely to experience harmful health effects during their lifetime in response to exposure to a particular metal in vegetal foods [17,28,29].

The total non-carcinogenic hazard attributable to ingestion of all metals within a vegetable was expressed as a multi-elemental risk-hazard index via the total target hazard quotient (*TTHQ*). It was calculated by summing the individual target hazard quotients for each metal analyzed, as shown below:

*TTHQ = ∑ THQi*; *i* = the number of analyzed metals

Related to interpreting this index, *TTHQ* < 1.0 shows minimal health impact, *TTHQ* value > 1.0 indicates potential health risks, and *TTHQ* > 10.0 denotes serious chronic risk [30].

### 2.6. Data Analysis

Levels of metals in non-cultivated plants and soils in which they are growing were analyzed with a one-way ANOVAs after checking the log-transformed data (decimal logarithmation) for normality and homogeneity of variances using Anderson–Darling tests and Bartlett’s tests, respectively. Post-hoc testing was run using the Scheffe’s procedure to adjust for the risk of type I error due to low sample size [31]. Our post-hoc analysis involved only pairwise comparisons between plant and soil metal contents, not all possible pairwise comparisons. A similar approach was used for samples collected from non-cultivated areas. We also applied ANOVA on data sets for spontaneous vegetation to identify whether there are differences among different locations in metal accumulation within the same plant species. All data are given as means with one standard deviation, and a *p* value ≤ 0.05 was considered significant. Statistical analysis was conducted with the software STATISTICA^®^ software (version 8).

## 3. Results and Discussions

### 3.1. Metal Levels and Pollution Assessment in Soils

**Figure 2:**
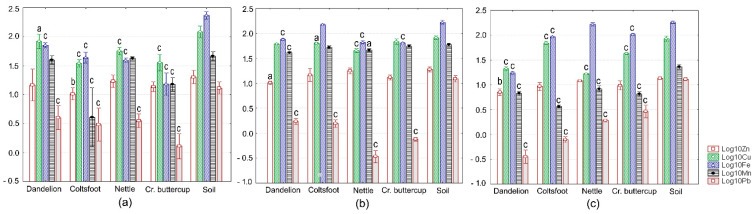
Measured values of Zn, Cu, Fe, Mn, and Mn levels in soils and aerial parts of the spontaneous plants examined. (**a**) Site S1; (**b**) Site S2; and (**c**) Site S3. Each value is the average for three replicates and is shown on a log_10_ scale as mean (vertical bar) with one standard deviation (error bar). Vertical bars marked with small letters indicate significant differences as compared to soil (Scheffe’s test, ^c^—*p* < 0.001, ^b^—*p* < 0.01, and ^a^—*p* < 0.05).

**Figure 3:**
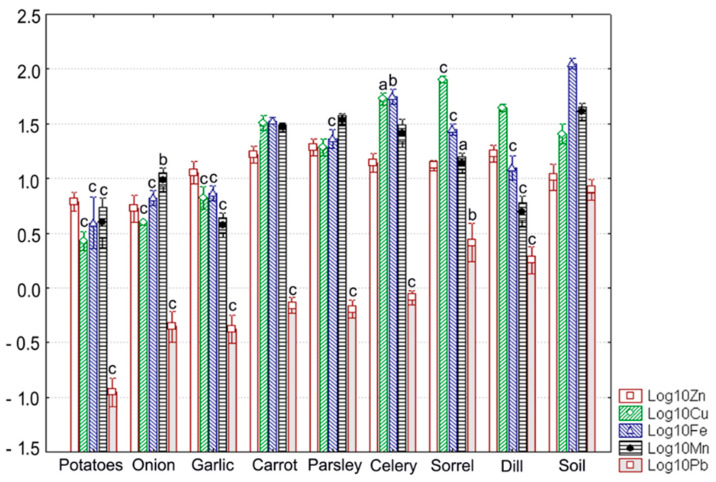
Measured values of Zn, Cu, Fe, Mn, and Pb levels in soils and vegetables at the reference site (site R). Each value is the average for three replicates and is shown on a log_10_ scale as mean (vertical bar) with one standard deviation (error bar). Vertical bars marked with small letters indicate significant differences as compared to soil (Scheffe’s test, ^c^-*p* < 0.001, ^b^-*p* < 0.01, ^a^-*p* < 0.05).

Concentrations of soil Zn, Cu, Fe, Mn, and Pb at different sites are given in Table 1, Figure 2a–c, and in Figure 3. Selected data on relevant levels of target metals in soils were compiled from environmental literature and are also presented in Table 1. We found that the measured values tended to decrease in the following order: Fe > Cu > Mn > Zn > Pb. This order of relative abundance is broadly similar to those from other environmental surveys conducted in the Bor area at sites with higher metal contents of soils [12,13,32]. These findings also reveal a substantial spatial variability of soil metal levels within a 30 km radius around RTB Bor [12,13,14,15].

Metal concentrations were within the normal range in surface soil horizons, with only Cu occurring at higher levels compared to the corresponding relative average global contents (Table 1, Figure 2a–c and Figure 3). The measured values for most target metals were lower than the phytotoxic levels and the benchmarks given in the relevant Serbian and European Directives (Table 1, Figure 2a–c and Figure 3). At sites S1, S2, and S3, soil Cu showed phytotoxic levels (Table 1, Figure 2a–c). With respect to the benchmark values set by the Serbian regulation related to soil quality, Cu was above the threshold value (TV) at sites S1, S2, and S3, and the maximum allowable concentration (MAC) at site S1 (Table 1, Figure 2a–c). Comparing soil metal levels determined in this study to such reference values approximately assesses the probability of contamination but cannot provide scientists with overall information about the status of soil quality.

Excepting Mn, the highest and the lowest amounts of target metals were found at site S1 and site R, respectively. These data confirm our choice of using site R as the reference location in terms of soil quality. Location of site S1 within the mine dumps from Open Pit Bor may also explain the presence of the highest soil metal concentrations at this location.

Our sampling points were distinct from those used in other studies investigating soil metal contamination in the Bor area (see Table 1). Related to the study of Nikolić et al. (2011), site S1 was close to two sites analyzed in this work, i.e., site 4 (General Hospital Bor) and site 8 (Šumska sekcija); while sites R, S2, and S3 lay at least 1.5 km away from the other ten sites used in the aforementioned work [12]. With respect to the other studies mentioned in Table 1 [13,14,15], the distance between the current sampling points and those used in the aforementioned investigations varied between 2 and 20 km. Metal contents observed here were within the lower limit of the concentration range reported at other sites affected by contamination originating from the RTB Bor [12,13,14,15,32] or from other non-ferrous mining operations located along the same copper-ore belt, such as Moldova Nouă in Romania [17] (Table 1, Figure 2a–c and Figure 3). In addition, these concentrations were markedly below those identified in other areas exposed to copper mining and smelting. For example, Meza-Figueroa et al. (2009) reported the following average metal levels in soils surrounding a former copper mine in Mexico: Zn—171.2 mg kg^−1^ dw, Cu—236.3 mg kg^−1^ dw, Fe—26,593.5 mg kg^−1^ dw, Mn—366 mg mg kg^−1^ dw, and Pb—32.8 mg mg kg^−1^ dw [38]. The values detected in the mine tailings at the El Teniente-Codelco copper mine in Chile were: Zn—76 mg kg^−1^ dw, Cu—1150 mg kg^−1^ dw, Fe—36,500 mg kg^−1^ dw, and Pb—76 mg kg^−1^ dw [39], whereas the values assessed for the abandoned copper-mine tailings at Mynydd Parys in the UK were: Zn—1036 mg kg^−1^ dw, Cu—1905 mg kg^−1^ dw, Fe—4229 mg kg^−1^ dw, and Pb—7692 mg kg^−1^ dw [40].

Mean values for the geo-accumulation index, the enrichment factor, and the Nemerow integrated pollution index from all sites are given in Table 2. All sites had *I_geo_* values for Zn, Fe, Mn, and Pb below 0, indicating the lack of contamination from these metals (Table 2). There was also no contamination with Cu at site R. In contrast, there was a low to moderate Cu contamination at the other sites examined, with the measured values for *I*_geo_ being above 1 (Table 2).

After using Fe as a reference metal for computing enrichment factors, we detected deficiency for Zn, Mn, and Pb at all sites (Table 2). In the case of Cu, the measured *EF* values revealed a moderate enrichment at sites S1, S2, and S3 (Table 2). When using a threshold value of 1.5 to delineate between metal concentrations derived from natural processes and other sources, such as point and non-point pollution [41], it is likely that a significant proportion of Cu found in soils collected from the three latter sites could originate from anthropogenic sources (Table 2). With respect to the multi-element contamination of soils, the application of Nemerow integrated pollution index revealed no pollution for site R, warning line of pollution for sites S2 and S3, and low level of pollution for site S1 (Table 2).

On the one hand, it is plausible to consider that our findings provide indication for a low-to-moderate contamination levels at sites S1, S2, and S3, with Cu potentially derived from human activities being the main contaminant of environmental concern (Table 2). This is in line with the results of previous studies attesting to the elevated copper contamination in soils from the Bor area ([12,13,14,15,32]. On the other hand, one could also expect to find, in the sites investigated here, higher background levels of natural copper not derived from human activities since the study area lies within a copper belt [17]. Therefore, the moderately increased values of pollution indices may not be associated only with anthropogenic activities; rather, they may reflect, at least to some degree, soil Cu levels normally encountered in this area.

These disparate conclusions may stem from the weaknesses of these indices. Thus, inappropriate choice of GB may lead to unrealistically high values for *I*_geo_. This could also be an issue when computing the *EF* values. In addition, both these indices do not take into account the natural geochemical variability. Moreover, *PI_N_* does not include a weighing factor, hence not allowing a ranking of elements based on their contribution to contamination/pollution.

### 3.2. Metal Levels in Plants

Concentrations of Zn, Cu, Fe, Mn, and Pb measured here in vegetal samples are shown in Figure 2a–c (for spontaneous plants), in Figure 3 (for vegetables), and in Table 3. Selected data on levels of target metals in different plants were compiled from specialty literature and are summarized in Table 4. The relative abundance of metals in aerial parts of spontaneous plant species was generally similar to that observed in the corresponding soils, more precisely: Fe > Cu > Mn > Zn > Pb. This trend is comparable to that observed by Antonijevic et al. (2011) in vegetation from the same area [14]. However, no clear pattern of variation in metal content was observed for food crops, which may be related to different plant parts used for chemical analysis. In addition, no evidence of phytotoxicity could be found from the levels of target metals in the vegetation collected at the four sites examined.

Descriptive statistics revealed different patterns of metal accumulation among different spontaneous plants (Figure 2a–c, Table 3). The mean Zn level was highest in nettle collected at site S2 and lowest in dandelion at site S3. Conversely, Cu showed the greatest and the smallest average amounts in dandelion at site S1 and in nettle at site S3, respectively. Fe displayed the widest range of concentration, with the maximum mean content observed in nettle at site S3 being more then 10-fold higher than the minimum mean content determined in creeping buttercup at site S1. Mn accumulated to its highest level in creeping buttercup at site S2, whereas the lowest level was seen in coltsfoot at site S3. Pb exhibited the narrowest range of concentrations, with a maximum average in dandelion at site S1 and minimum average in nettle at site S2, respectively. These metal levels were within the normal range for Zn, Fe, Mn, and Pb, but they fell within the critical range for Cu (Table 3 and Table 4).

Nettle and dandelion serve as valuable phyto-indicators of environmental pollution [46], but few data are available on metal levels in plant specimens collected from the Bor area. Mijatović et al. (2018) investigated accumulation of Zn, Cu, and Pb in the aforementioned plants at six sites near the RTB Bor. The measured values in leaves were comparable for Cu but higher for Zn and Pb [45] (Figure 2a–c, Table 3). Several other studies have examined metal retention in spontaneous vegetation collected from this area. For example, Antonijevic et al. (2012) investigated Zn, Cu, Fe, Mn, and Pb concentrations in leaves and stems of other relevant phyto-indicators, including brown knapweed (*Centaurea jacea* L., 1753; fam. *Asteraceae*), orange mullein (*Verbascum phlomoides* L., 1753; fam. *Scrophulariaceae*), and soapwort (*Sapponaria officinalis* L., 1753; fam. *Caryophyllaceae*). These plants were obtained from 16 sites located near the old flotation tailing ponds of RTB Bor. It was found that metal levels were generally higher than those determined in the present work [14] (Figure 2a–c, Table 3 and Table 4). Given substantial differences in metal concentrations between different plant parts or closely related species [1], such comparisons should be treated with caution, or preferably, avoided. However, all these plants are routinely consumed by primary consumers, such as insects and snails [47,48], which serve as key mediators of metal transfer along terrestrial food chains. These findings provide pertinent evidence for a relatively low ecotoxicological risk for sites analyzed in this work.

Statistical analysis revealed significant intersite differences in metal levels of non-cultivated plants (ANOVA, *p* ≤ 0.012), except for Zn in coltsfoot and creeping buttercup (ANOVA, *p* ≥ 0.062). However, we neither found any clear pattern of metal variation in different plant specimens across different sites nor saw a similarity between intersite metal variation in different plant species (Figure 2a–c). This highlights the difficulties in using metal content of plant tissues or species-specific patterns of metal content for the evaluation of ecotoxicological hazards and risks.

With respect to food crops (as shown in Figure 3 and Table 4), the average Zn content was lowest in onion bulbs and highest in carrot roots. Cu exhibited the widest concentration range, with maximum and minimum levels being determined in potatoes (2.69 ± 0.55 mg kg^−1^ dw) and sorrel leaves, respectively. A similar trend was observed for Pb; the limits of the range of average Pb content were observed in potatoes and sorrel leaves. Fe occurred again at the lowest level in potatoes (4.33 ± 2.24 mg kg^−1^ dw) but reached the maximum level in celery roots. In contrast, the minimum mean concentration of Mn was detected in garlic bulbs, whereas the maximum mean concentration was seen in parsley roots.

Levels of Zn, Mn, and Fe in cultivated plants were below the maximum permissible concentrations recommended by WHO/FAO (Figure 3, Table 3 and Table 4). By contrast, these guideline values were exceeded for Cu in leafy vegetables (dill, sorrel) and celery roots (Figure 3, Table 3 and Table 4). Pb contents of most vegetables, excluding potatoes, were also well above the safe limit suggested by WHO/FAO (0.3 mg kg^−1^ dw). The highest values were determined in leafy vegetables, with the measured values in sorrel almost reaching the maximum permissible concentration in dry vegetables set by the Serbian law to 3 mg kg^−1^ dw [49]; that is 10-fold higher than the safe limit as per WHO/FAO (Table 4).

Several authors reported similar Cu and Pb concentrations in common vegetables grown on soils contaminated by non-ferrous metal mining and processing [17,50,51,52]. For example, Zhuang et al. (2009) found levels from 5.0 up to 14.3 for Cu and from 0.90 up to 2.23 for Pb, respectively, in vegetables collected near the Dabaoshan polymetallic mine in Southern China [53]. Wu et al. (2011) reported Cu levels varying between 5.9 and 9.7 mg kg^−1^ dw in bulb vegetables (onion, garlic) grown in the vicinity of the Jiuhuashan copper mine [50]. Notwithstanding the differences in reporting metal levels, one can see that comparable values were also determined for common vegetables from the old copper mining area of Moldova Noua [17,21] located about 50 km away from the RTB Bor, along the same copper-ore belt (Figure 3, Table 3 and Table 4).

However, Pb occurrence at elevated levels in vegetables may be related not only to non-ferrous ore processing, but also to other major anthropogenic sources, such as contamination from vehicle exhausts or sewage sludge use in agriculture [2]. In this context, it is worth mentioning that Pajevićet et al. (2018) reported average Pb concentrations ranging between 1.11 and 3.56 mg kg^−1^ dw in vegetables grown at different locations in the Vojvodina Province [49]. Similarly, Arsenov et al. (2016) determined levels up to 6 mg kg^−1^ dw in vegetables from the green markets in Novi Sad (Serbia) [54].

### 3.3. Metal Trasfer from Soil to Plants

The log_10_-transformed data sets for wild plants and soils in which they are growing were normally distributed (Anderson–Darling test, *p* ≥ 0.125) and homoscedastic (Bartlett’s test, *p* ≥ 0.053). Similar results were obtained when analyzing the metal transfer from soil to food crops (Anderson–Darling test, *p* ≥ 0.085; Bartlett’s test, *p* ≥ 0.057). For spontaneous vegetation, ANOVA yielded significant results, irrespective of sites and metals examined (*p* ≤ 0.039). Post-hoc analyses using the Scheffe’s approach mainly showed significant decreases in metal content of non-cultivated plants versus the soil (Figure 2a–c). This trend was most consistent in dandelion and for Cu and Pb (Figure 2a–c).

Application of ANOVA showed significant differences in metal content of vegetal foodstuffs and the corresponding soil (*p* ≤ 0.002). Post-hoc pairwise comparisons using the Scheffe’s method often revealed significant reductions in levels of metals in cultivated plants relative to the values measured in the soil (Figure 3). This trend was particularly evident for Pb (Figure 3). It was also found for Fe and Mn, except for bulb vegetables (onion and garlic) and root vegetables (carrot, parsley, and celery), respectively. However, there were no significant differences in the uptake of Zn, irrespective of the plant analyzed (Figure 3). In contrast, Cu displayed a bidirectional response, occurring at significantly elevated levels in celery roots and sorrel leaves and at significantly lower levels in potatoes and bulb vegetables, i.e., onion and garlic (Figure 3). Among vegetables, the most homogeneous response was observed for potatoes, with all target metals occurring at significantly lower concentrations compared to the soil (Figure 3).

Based on levels to which contaminants accumulate in their aboveground tissues, plants can be classified into three categories: accumulators/hyperaccumulators, indicators, and metal excluders [1,2]. The latter category includes species that are able to maintain low levels of metals in their aerial parts compared to those measured in soils. This is consistent with the pattern identified for all metals in aerial parts of spontaneous plant species, as well as in dill leaves. In contrast, sorrel leaves showed a significant increase in Cu levels compared to the soil, suggesting that this species may act as an indicator or accumulator for this element. Indeed, several studies attest to the ability of this plant species and other *Polygonaceae* to absorb, tolerate, and accumulate copper; and to maintain normal growth in Cu-contaminated environments [55,56,57]. However, metal accumulation in plants is a complex process that depends not only on soil metal levels and vegetal tissue analyzed, but also on factors like redox potential of soil, amount of organic matter, soil pH, and rate of metal addition into soil [1,2,31,32,37]. Such parameters should be measured in future studies aiming to conduct ecotoxicological and human health risk assessments for the Bor area.

### 3.4. Health Risk Assessment

The present study has taken into account for human health risk assessment only the gastrointestinal route related to food consumption. The gastrointestinal route associated to metal exposure via drinking water or via absorption through skin was not considered. The risk assessment for non-carcinogenic health effects associated with consumption of vegetables grown in the Bor area was performed using the target hazard quotients (THQs) and the total target hazard quotients (TTHQs). These formula-derived measurements provide scientists with an cost-effective and rigorous indication of risk related to long-term exposure to chemical pollutants using known safe-dose limits [58].

**Figure 4:**
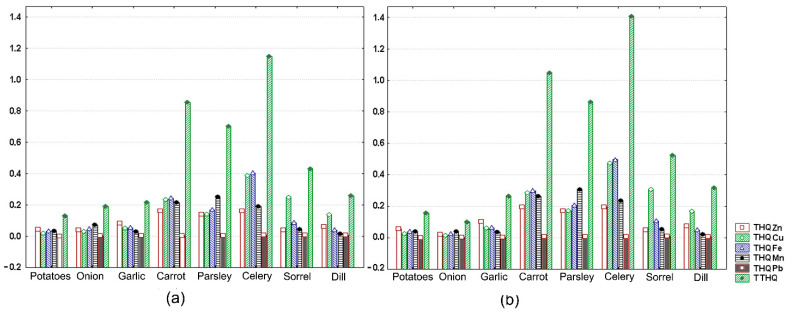
Measured values for THQ_i_ of Zn, Cu, Fe, Mn, and Mn and for TTHQ in vegetables collected from site R. (**a**) male (**b**) female. Each value is the mean for three replicates and is represented by a vertical bar (column).

Values of this parameter for vegetables investigated are illustrated in Figure 4a,b and given as absolute values in Table 5. Individual THQs were well below 1 for all metals, irrespective of plant foodstuff analyzed. The highest values were found for Cu in celery roots (0.47 for female and 0.39 for male), carrot roots (0.28 for female and 0.23 for male), and sorrel leaves (0.30 for female and 0.25 for male); for Fe in celery roots (0.50 for female and 0.41 for male) and carrot roots (0.30 for female and 0.24 for male); and for Mn in parsley roots (0.31 for female and 0.25 for male) and carrot roots (0.26 for female and 0.21 for male). This trend of root vegetables (carrot, parsley, and celery) showing higher THQs for the aforementioned metals than leafy vegetables (dill, sorrel) is in agreement with the results of other observational studies investigating metal accumulation in vegetal foods grown in old copper-mining areas [17,21]. Since exposure levels estimated here via THQ are less than 1, it is also expected that daily of exposure to single metals in vegetables collected from the reference site is unlikely to induce adverse health effects during a person’s life time.

Despite all individual THQs being below 1, the measured values for the combined index TTHQ approached or surpassed 1 for celery roots and carrot roots in both females (1.41; 1.05) and males (1.15; 0.85), with the main contributors being Cu and Fe. Similar trends for TTHQ, with Cu and/or Fe dominance were reported for other copper-mining areas [21,52,59,60]. Although these THQs are greater than 1.0, they are relatively low and do not imply that adverse health effects will occur. However, based on the same values, one cannot totally exclude a potential long-term risk of non-carcinogenic effects associated with dietary consumption of these vegetables although they originate from a relatively unpolluted area. These data warrant further evaluation, especially related to the combined effect of Cu and Fe. This could be of particular interest in the context of rapid population aging in Serbia and worldwide, since both these essential metals, when found in excess and/or acting synergically, could contribute to diseases like atherosclerosis and neurodegenerative diseases [61].

Thus, trace small or moderate amounts of copper (<1 mg kg^−1^ dw) in drinking water can substantially increase the risk of neurodegenerative diseases in animal models [61]. In fact, free copper levels in the human brain as low as 0.12 mg kg^−1^ dw were found to impede the efflux of amyloid from the brain, thus promoting formation of amyloid plaques, one of the hallmarks of Alzheimer’s disease [61]. There is also epidemiological evidence correlating some measure of atherosclerosis with some measure of iron stores (serum transferrin or ferritin saturation) and copper stores (blood copper or ceruloplasmin [61].

As with the majority of environmental health investigations, the present study is subject to some limitations. The first limitation is the fact that sampling activities were performed in triplicates in only four locations. It was therefore not possible to provide a more detailed insight into the spatial change characteristics of soil pollution/contamination with heavy metals in the Bor area. Yet, such approaches are regularly used in exploratory studies of environmental pollution, such is the case of our work, due various reasons, including costs and insufficient properly trained personnel.

Second, for both Cu and Fe, the RfD values were extrapolated from the US maximum contaminant level goal (MCLG) for drinking water, which relies on acute gastrointestinal effects of these metals [25]. This approach is regularly used in environmental literature as a source of toxicity factors. However, there are currently many widely accepted oral RfD values for these essential elements given the complexity of assessing limits for their intake due to their U-shaped dose-response curve [2,62]. As a result, the health effects corresponding to the use of this derivation for oral exposure may differ from those reported for exposure via drinking water.

Future studies are required not only to expand on the ecotoxicological and human health significance of our results but also to refine our knowledge of metal transfer along terrestrial food chains in the Bor area. These studies need to be performed with a larger sample size and must be designed to include more plant species, locations, and soil parameters. Such investigations should also be extended to cereals and animal foodstuffs for gaining an in-depth biological insight on the health risks related to consumption of vegetal foodstuffs grown in this area. It would also be of interest to investigate potential cancer effects associated with consumption of metal-contaminated vegetables collected from this area. To this end, we plan to sample the local cancer population for future studies and compare the metal levels in vegetal plants with the current results. In this context, we do believe that this kind of fundamental work can be applied to strengthen the scientific basis of local government decisions aiming to reduce the environmental and human risks in areas with a long history of metal pollution/contamination.

## 4. Conclusions

- The highest and the lowest amounts of soil Zn, Cu, Fe, Mn, and Pb were regularly found at site S1 and site R, respectively. The measured values were below the benchmarks given in the relevant Serbian and European Directives, except for Cu. This metal was found at concentrations above the maximum allowable concentration (MAC) for Serbia at site S1, as well as above the threshold value (TV) for Serbia at sites S2 and S3. Application of different pollution indices revealed low-to-moderate contamination levels at sites S1, S2 and S3, with Cu—likely deriving from anthropogenic activities—being the main contaminant of environmental concern.
- No evidence of phytotoxicity could be found from the levels of Zn, Cu, Fe, Mn, and Pb in the spontaneous vegetation collected, indicating a relatively low ecotoxicological risk for sites analyzed in this work. The safe limit suggested by WHO/FAO for Cu (40 mg kg^−1^ dw) and Pb (0.3 mg kg^−1^ dw) in plant foodstuffs were respectively exceeded in leafy vegetables (dill, sorrel) and celery roots, and in most vegetables, excluding potatoes.
- Metal concentrations in aerial parts of spontaneous plant species were often significantly lower than the values determined in the corresponding soils. This trend was most consistent in dandelion and for Cu and Pb. Significantly reduced Pb, Fe, and Mn also tended to occur in vegetables. In contrast, Cu was found at significantly elevated levels in celery roots and sorrel leaves, whereas Zn tended to show similar values to that seen in soils.
- Assessment of non-carcinogenic risk associated with consumption of vegetables grown in relatively unpolluted soils from the Bor area was conducted using the current methodology (US EPA–WHO FAO) via calculating the target hazard quotients (THQs) and the total target hazard quotients (TTHQs), with RfDs for most metals being based on gastrointestinal tract irritation. It was found that consumption of celery roots and carrot roots in the case of females (1.41; 1.05) and carrot roots in the case of males (1.15; 1.02) might pose a long-term risk of non-carcinogenic effects, with the main potential contributors being Cu and Fe.
- Expansion of present research to other plant species (cereals, fruits, and other vegetables), locations, and routes of metal exposure, e.g., consumption of animal foods (meat, milk, and eggs), drinking water, or contact with air, is imperative for enlarging our understanding of populational risk associated with Cu mining and processing in the Bor area specifically, and other areas with a long history of non-ferrous metal pollution in general.

## Figures and Tables

**Table 1:** Selected data on relevant levels of target metals in soils (mg kg^−1^ dw).

	Zn	Cu	Fe	Mn	Pb	Reference
Concentration range in surface horizons (worldwide)	17–125	1–140	1000–100,000	7–9200	1.5−176	[1]
Mean concentration in surface horizons (worldwide)	64	14	45,000	437	25	[2]
Mean level in upper continental crust	67	28	-	438.59	17	[33]
	71	25	-	-	17	[34]
Limit values in soils as per European Council Directive 86/278/EEC	150–300	50–140	50–300	-	-	[35]
Threshold values (TV) for Serbia	140	36	-	-	85	[36]
Maximum allowable concentrations (MAC) for Serbia	300	100	-	-	100	[36]
Intervention values (IV) for Serbia	720	190	-	-	530	[36]
Site R	10.51	25.97	112.24	41.01	7.88	
Site S1	20.24	122.01	234.92	46.20	3.27	
Site S2	19.15	82.04	167.31	59.02	12.47	
Site S3	23.53	84.99	182.07	23.18	12.94	
Bor (16 sites)	40–890 (240.1)	440–3800 (1558.6)	16,200–58,200 (29,462.5)	55–900 (459.9)	21–1700 (264.1)	[14]
Bor (8 sites)	43.4–465	9.2–1586	10,010–19,245	-	21.99–238.4	[15]
Bor (12 sites)	-	220–2540 (913.33)	-	460–1300 (1070)	40–230 (86.67)	[12]
Bor (5 sites)	72–126	84–408	-	-	5.8–57.5	[13]
Moldova Nouă	197.03	229.15	44,582.45	1,845.28	22.02	[17]
Phytotoxic values	100–500	36–698	-	-	100–500	[37]

-, no relevant data. Values in parentheses denote average values.

**Table 2:** Measured values for selected pollution indices.

		Zn	Cu	Fe	Mn	Pb
*I*_geo_	Site S1	−2.32	1.86	−8.17	−3.83	−1.18
	Site S2	−2.40	1.29	−8.66	−3.47	−3.59
	Site S3	−2.90	1.34	−8.53	−4.82	−3.30
	Site R	−3.26	−0.37	−9.23	−4.00	−1.14
*EF*	Site S1	0.22	4.05		0.08	0.49
	Site S2	0.30	3.82		0.14	0.66
	Site S3	0.19	3.64		0.05	0.63
	Site R	0.24	1.80		0.15	0.62
*Pi*	Site S1	0.30	5.46	0.005	0.11	0.66
	Site S2	0.28	3.67	0.004	0.14	0.63
	Site S3	0.20	3.81	0.004	0.05	0.66
	Site R	0.16	1.16	0.002	0.09	0.40
*PI_N_*	Site S1	1.12				
	Site S2	0.76				
	Site S3	0.78				
	Site R	0.24				

*I*_geo_, the geo-accumulation index; *EF*, the enrichment factor; *Pi*, the single pollution index of each metal; *PI_N_*, the Nemerow integrated pollution index.

**Table 3:** Measured levels of target metals in plants (mg kg^−1^ dw).

	Zn	Cu	Fe	Mn	Pb
Dandelion (site S1)	15.04 (3.51)	83.38 (9.46)	71.05 (3.08)	39.87 (2.69)	4.04 (0.81)
Dandelion (site S2)	10.22 (0.86)	61.05 (1.58)	75.89 (3.16)	41.17 (1.53)	1.72 (0.25)
Dandelion (site S3)	7.18 (1.10)	21.23 (2.01)	17.28 (1.43)	6.76 (0.73)	0.37 (0.11)
Coltsfoot (site S1)	10.36 (1.01)	34.84 (1.95)	44.01 (3.89)	4.36 (1.77)	3.12 (0.75)
Coltsfoot (site S2)	15.15 (4.63)	63.41 (2.31)	148.52 (6.18)	52.67 (4.19)	1.58 (0.30)
Coltsfoot (site S3)	9.43 (1.87)	69.39 (6.43)	92.59 (2.99)	3.65 (0.32)	0.79 (010)
Nettle (site S1)	17.07 (1.65)	56.69 (3.28)	39.48 (1.33)	42.34 (1.19)	3.54 (0.38)
Nettle (site S2)	17.90 (2.36)	45.19 (4.39)	66.15 (5.58)	46.03 (3.10)	0.35 (0.11)
Nettle (site S3)	12.04 (0.34)	16.64 (0.39)	166.28 (12.91)	8.37 (1.20)	1.90 (0.13)
Creeping buttercup (site S1)	13.76 (1.06)	35.64 (4.62)	15.46 (2.62)	15.11 (1.59)	1.30 (0.26)
Creeping buttercup (site S2)	13.17 (1.75)	68.45 (8.79)	63.90 (2.11)	55.98 (2.88)	0.75 (0.12)
Creeping buttercup (site S3)	9.85 (2.17)	42.65 (2.41)	103.29 (5.49)	6.58 (0.81)	2.99 (0.84)
Potatoes tubers (site R)	6.22 (1.31)	2.69 (0.55)	4.33 (2.24)	4.36 (2.44)	0.11 (0.01)
Onion bulbs (site R)	5.48 (1.72)	3.94 (0.27)	6.68 (1.17)	9.96 (2.48)	0.45 (0.16)
Garlic bulbs (site R)	11.53 (2.59)	6.76 (1.77)	7.40 (1.23)	3.82 (0.99)	0.43 (0.14)
Carrot roots (site R)	22.30 (4.16)	32.39 (5.50)	33.74 (2.43)	29.63 (2.67)	0.69 (0.11)
Parsley roots (site R)	19.35 (3.48)	19.48 (3.82)	23.41 (4.89)	34.63 (5.04)	0.64 (0.12)
Celery roots (site R)	22.14 (4.61)	53.69 (7.33)	56.28 (9.15)	26.55 (7.48)	0.82 (0.13)
Dill leaves (site R)	13.21 1.47)	44.34 (3.73)	28.10 (3.41)	14.04 (2.96)	2.73 (1.05)
Sorrel leaves (site R)	19.88 (3.77)	25.97 (5.31)	12.75 (3.56)	5.17 (1.62)	1.84 (0.53)

Values are shown as average concentrations with one standard deviation (in parentheses).

**Table 4:** Selected literature data on relevant levels of target metals in plants (mg kg^−1^ dw).

	Zn	Cu	Fe	Mn	Pb	Reference
Normal range in plants	1–400	5–20	40–500	20–1000	0.2–20	[37,42,43,44]
Critical values in plants	100–400	20–100	-	30–500	30–300	[37,42,43,44]
Phytotoxic values	400	200	-	3000	100–500	[1]
Common dandelion (leaves)	28.8–90.3	42.9–192.5	-	-	2.7–17.8	[45]
Nettle (leaves)	32.1–60.8	85.9–171.6	-	-	3.4–17.3	
Soapwort (leaves)	800	350	100	23	18	[14]
Soapwort (stems)	280	150	90	10	9	
Orange mullein (leaves)	1400	630	130	25	1400	
Orange mullein (stems)	590	70	30	10	590	
Brown knapweed (leaves)	380	200	60	14	9	
Brown knapweed (stems)	390	70	30	5	8	
Parsley roots	7.74 *	6.88 *	99.02 *	8.10 *	0.66 *	[17]
Carrot roots	3.18 *	1.77 *	31.89 *	2.23 *	0.09 *	
Bulb onions	2.01 *	1.37 *	4.65 *	1.34 *	0.13 *	
Cabbage leaves	8.51 *	2.77 *	31.53 *	9.15 *	0.25 *	
Letuce leaves	5.14 *	2.22 *	16.90 *	4.12 *	0.21 *	
Parsley leaves	10.44	4.79 *	106.75 *	9.72 *	0.50 *	
Potatoes (tuberized roots)	4.14	7.36 *	10.42 *	3.92 *	0.45 *	[21]
FAO	60	40	450	500	0.30	[44]

-, no relevant data; *, values expressed as mg kg^−1^ fresh weight.

**Table 5:** Measured levels of individual target hazard quotients (THQs) and the total target hazard quotients (TTHQs).

Sex	Vegetal Food	THQ (Zn)	THQ (Cu)	THQ (Fe)	THQ (Mn)	THQ (Pb)	Sum THQ
Male	Potatoes tubers	0.04	0.02	0.03	0.03	0.00	0.13
	Onion bulbs	0.04	0.03	0.05	0.07	0.00	0.19
	Garlic bulbs	0.08	0.05	0.05	0.03	0.00	0.22
	Carrot roots	0.16	0.23	0.24	0.21	0.01	0.85
	Parsley roots	0.14	0.14	0.17	0.25	0.00	0.70
	Celery roots	0.16	0.39	0.41	0.19	0.01	1.15
	Dill leaves	0.04	0.25	0.09	0.04	0.01	0.43
	Sorrel leaves	0.06	0.14	0.04	0.02	0.01	0.26
Female	Potatoes tubers	0.05	0.02	0.04	0.04	0.00	0.16
	Onion bulbs	0.02	0.01	0.03	0.04	0.00	0.10
	Garlic bulbs	0.10	0.06	0.07	0.03	0.00	0.26
	Carrot roots	0.20	0.28	0.30	0.26	0.01	1.05
	Parsley roots	0.17	0.17	0.21	0.31	0.01	0.86
	Celery roots	0.20	0.47	0.50	0.23	0.01	1.41
	Dill leaves	0.05	0.30	0.11	0.05	0.01	0.52
	Sorrel leaves	0.08	0.17	0.05	0.02	0.01	0.32

## Data Availability

Not applicable.

## References

[1] Kabata-Pendias A., Pendias H. (2001). Trace Elements in Soils and Plants.

[2] Kabata-Pendias A., Mukherjee A.B. (2007). Trace Elements from Soil to Human.

[3] Drăghici G.A., Dehelean C., Pinzaru I., Bordean D.M., Borozan A., Tsatsakis A.M., Kovatsi L., Nica D. (2019). Soil copper uptake by land snails: A semi-field experiment with juvenile Cantareus aspersus snails. Environ. Toxicol. Pharmacol..

[4] Zhang W., Liu M., Li C. (2020). Soil heavy metal contamination assessment in the Hun-taizi River watershed, China. Sci. Rep..

[5] Gall J.E., Boyd R.S., Rajakaruna N. (2015). Transfer of heavy metals through terrestrial food webs: A review. Environ. Monit. Assess..

[6] Benvenuto M.A. (2016). Metals and Alloys: Industrial Applications.

[7] FDA (Food and Drug Administration) (2001). Dietary Reference Intakes for Vitamin A, Vitamin K, Arsenic, Boron, Chromium, Copper, Iodine, Iron, Manganese, Molybdenum, Nickel, Silicon, Vanadium, and Zinc. Dietary Supplements.

[8] Filimon M.N., Nica D.V., Ostafe V., Bordean D.M., Borozan A.B., Vlad D.C., Popescu R. (2013). Use of enzymatic tools for biomonitoring inorganic pollution in aquatic sediments: A case study (Bor, Serbia). Chem. Cent. J..

[9] (2019). Pollution and Health Metrics: Global, Regional, and Country Analysis. https://gahp.net/wp-content/uploads/2019/12/PollutionandHealthMetrics-final-12_18_2019.pdf.

[10] Urošević S., Vuković M., Pejčić B., Štrbac N. (2018). Mining-metallurgical sources of pollution in Eastern Serbia and environmental consciousness. Rev. Int. Contam. Ambient..

[11] Šerbula S.M., Antonijević M.M., Milošević N.M., Milić S.M., Ilić A.A. (2010). Concentrations of particulate matter and arsenic in Bor (Serbia). J. Hazard. Mater..

[12] Nikolić Đ., Milošević N., Živković Ž., Mihajlović I., Kovačević R., Petrović N. (2011). Multi-criteria analysis of soil pollution by heavy metals in the vicinity of the Copper Smelting Plant in Bor (Serbia). J. Serb. Chem. Soc..

[13] Stanojlović R.D., Sokolović J.M., Milosević N. (2014). Integrated environmental protection and waste minimization in the area of Copper Mine Bor, Serbia. Environ. Eng. Manag. J..

[14] Antonijević M.M., Dimitrijević M.D., Milić S.M., Nujkić M.M. (2012). Metal concentrations in the soils and native plants surrounding the old flotation tailings pond of the Copper Mining and Smelting Complex Bor (Serbia). J. Environ. Monit..

[15] Dimitrijević M.D., Nujkić M.M., Alagić S.Č., Milić S.M., Tošić S.B. (2016). Heavy metal contamination of topsoil and parts of peach-tree growing at different distances from a smelting complex. Int. J. Environ. Sci. Technol..

[16] Nica D.V., Bordean D.M., Pet I., Pet E., Alda S., Gergen I. (2013). A novel exploratory chemometric approach to environmental monitorring by combining block clustering with Partial Least Square (PLS) analysis. Chem. Cent. J..

[17] Harmanescu M., Alda L.M., Bordean D.M., Gogoasa I., Gergen I. (2011). Heavy metals health risk assessment for population via consumption of vegetables grown in old mining area; a case study: Banat County, Romania. Chem. Cent. J..

[18] Kang Z., Wang S., Qin J., Wu R., Li H. (2020). Pollution characteristics and ecological risk assessment of heavy metals in paddy fields of Fujian province, China. Sci. Rep..

[19] Nujkić M.M., Dimitrijević M.M., Alagić S.Č., Tošić S.B., Petrović J.V. (2016). Impact of metallurgical activities on the content of trace elements in the spatial soil and plant parts of *Rubus fruticosus* L.. Environ. Sci. Process Impacts.

[20] Ahamad M.I., Song J., Sunm H., Wang X., Mehmood M.S., Sajid M., Su P., Khan A.J. (2020). Contamination level, ecological risk, and source identification of heavy metals in the hyporheic zone of the Weihe River, China. Int. J. Environ. Res. Public Health.

[21] Manea D.N., Ienciu A.A., Ştef R., Şmuleac I.L., Gergen I.I., Nica D.V. (2020). Health risk assessment of dietary heavy metals intake from fruits and vegetables grown in selected old mining areas-a case study: The Banat area of southern Carpathians. Int. J. Environ. Res. Public Health.

[22] United States Environmental Protection Agency (USEPA) (2000). Risk-Based Concentration Table.

[23] Life Expectancy at Birth, Total (Years)–Serbia. https://data.worldbank.org/indicator/SP.DYN.LE00.IN.

[24] World Health Organization (WHO) (1998). Health guideline for the use of wastewater in agriculture and aquaculture. Report of WHO science group, world health organization, Geneva, Switzerland. Technol. Rep. Sci..

[25] United States Environmental Protection Agency (USEPA) (2012). EPA Region III Risk-Based Concentration (RBC) Table 2008 Region III.

[26] United States Environmental Protection Agency (USEPA) (2011). USEPA Regional Screening Level (RSL) Summary Table.

[27] Average Sizes of Men and Women. https://www.worlddata.info/average-bodyheight.php.

[28] Naughton D.P., Petróczi A. (2008). Heavy metal ions in wines: Meta-analysis of target hazard quotients reveals health risks. Chem. Centr. J..

[29] Gruszecka-Kosowska A. (2019). Potentially harmful element concentrations in the vegetables cultivated on arable soils, with human health-risk implications. Int. J. Environ. Res. Public Health.

[30] Li X., Li Z., Lin C.J., Bi X., Liu J., Feng X., Zhang H., Chen J., Wu T. (2018). Health risks of heavy metal exposure through vegetable consumption near a large-scale Pb/Zn smelter in central China. Ecotoxicol. Environ. Saf..

[31] Cobb G.P., Sands K., Waters M., Wixson B.G., Dorward-King E. (2000). Accumulation of heavy metals by vegetables grown in mine wastes. Environ. Toxicol. Chem..

[32] Petrović J., Alagić S.Č., Milić S.M., Tošić S.B., Bugarin M.M. (2020). Chemometric characterization of heavy metals in soils and shoots of the two pioneer species sampled near the polluted water bodies in the close vicinity of the copper mining and metallurgical complex in Bor (Serbia): Phytoextraction and biomonitoring contexts. Chemosphere.

[33] Rudnick R.L., Gao S. (2003). Composition of the continental crust, treatise on geochemistry. Treatise Geochem..

[34] McLennan S.M. (2001). Relationships between the trace element composition of sedimentary rocks and upper continental crust. Geochem. Geophys..

[35] (1986). Commission of the European Communities Council Directive 12 on the protection of the environment, and in particular soil, when sewage sludge is used in agriculture. Off. J. Eur. Commun. L.

[36] (2018). Regulation on limit values of polluting, harmful and dangerous substances in soil. Off. Gazette RS.

[37] Alloway B.J., Alloway B.J. (2011). Sources of heavy metals and metalloids in soils. Metals in Soils: Trace Metals and Metalloids in Soils and Their Bioavailability.

[38] Meza-Figueroa D., Maier R.M., de la O-Villanueva M., Gomez-Alvarez A., Moreno-Zazueta A., Rivera J., Campillo A., Grandlic C.J., Anaya R., Palafox-Reyes J. (2009). The impact of unconfined mine tailings in residential areas from a mining town in a semi-arid environment: Nacozari, Sonora, Mexico. Chemosphere.

[39] Hansen H.K., Yianatos J.B., Ottosen L.M. (2005). Speciation and leachability of copper in mine tailings from porphyry copper mining: Influence of particle size. Chemosphere.

[40] Khan M.J., Jones D.L. (2008). Chemical and organic immobilization treatments for reducing phytoavailability of heavy metals in Coppermine tailings. J. Plant Nutr. Soil Sci..

[41] Barbieri M. (2016). The importance of enrichment factor (EF) and geoaccumulation index (Igeo) to evaluate the soil contamination. J. Geol. Geophys..

[42] Mukhopadhyay S., Maiti S.K. (2010). Phytoremediation of metal enriched mine waste: A review. Glob. J. Environ. Res..

[43] Kastori R. (1997). Heavy Metal in the Environment.

[44] WHO/FAO (2007). Expert Committee on Food Additives.

[45] Mijatović N., Pezo L., Terzić A., Šerbula S., Kovačević R. (2018). The biometrics techniques for the assessment of the degree of adoption of toxic and essential elements. Zašt. Mater..

[46] Gjorgieva D., Kadifkova-Panovska T., Bačeva K., Stafilov T. (2011). Assessment of heavy metal pollution in Republic of Macedonia using a plant assay. Arch. Environ. Contam. Toxicol..

[47] Dallinger R., Berger B., Triebskorn- Köhler R., Köhler H., Barker G.M. (2001). Soil biology and ecotoxicology. The Biology of Terrestrial Molluscs.

[48] Nica D.V., Bordean D.M., Borozan A.B., Gergen I., Bura M., Banatean-Dunea I. (2012). Use of land snails (*Pulmonata*) for monitoring copper pollution in terrestrial ecosystems. Rev. Environ. Contam. Toxicol..

[49] Pajević S., Arsenov D., Nikolić N., Borišev M., Orčić D., Župunski M., Mimica-Dukić N. (2018). Heavy metal accumulation in vegetable species and health risk assessment in Serbia. Environ. Monit. Assess..

[50] Wu F., Liu Y., Xia Y., Shen Z., Chen Y. (2011). Copper contamination of soils and vegetables in the vicinity of Jiuhuashan copper mine, China. Environ. Earth Sci..

[51] Xu D., Zhou P., Zhan J., Gao Y., Dou C., Sun Q. (2013). Assessment of trace metal bioavailability in garden soils and health risks via consumption of vegetables in the vicinity of Tongling mining area, China. Ecotoxicol. Environ. Saf..

[52] Pipoyan D., Beglaryan M., Sireyan L., Merendino N. (2019). Exposure assessment of potentially toxic trace elements via consumption of fruits and vegetables grown under the impact of Alaverdi’s mining complex. Hum. Ecol. Risk Assess..

[53] Zhuang P., Zou B., Li N.Y., Li Z.A. (2009). Heavy metal contamination in soils and food crops around Dabaoshan mine in Guangdong, China: Implication for human health. Environ. Geochem. Health.

[54] Arsenov D.D., Nikolić N.P., Borišev M.K., Župunski M.D., Pajević S.P. (2016). Heavy metal contamination of vegetables from green markets in Novi Sad. Zb. Matice Srp. Prir. Nauke.

[55] Jiang L., Shi W., Yang X., Fu C., Chen W. (2002). Cu-hyperaccumulators in mining area. Ying Yong Sheng Tai Xue Bao.

[56] Li H., Tang S.R., Heng J.M. (2005). Cu tolerance and accumulation of *Rumex acetosa* Linn, *Polygonum microcephalum* D. Don and *Rumex hastatus* D. Don. Bull. Sci. Technol..

[57] Yashim Z.I., Kehinde I.O., Hannatu M.A. (2014). Study of the uptake of heavy metals by plants near metal-scrap dumpsite in Zaria, Nigeria. J. Appl. Chem..

[58] Guo B., Hong C., Tong W., Xu M., Huang C., Yin H., Lin Y., Fu Q. (2020). Health risk assessment of heavy metal pollution in a soil-rice system: A case study in the Jin-Qu Basin of China. Sci. Rep..

[59] Tepanosyan G., Sahakyan L., Belyaeva O., Asmaryan S., Saghatelyan A. (2018). Continuous impact of mining activities on soil heavy metals levels and human health. Sci. Total Environ..

[60] Tepanosyan G., Sahakyan L., Maghakyan N., Saghatelyan A. (2020). Combination of compositional data analysis and machine learning approaches to identify sources and geochemical associations of potentially toxic elements in soil and assess the associated human health risk in a mining city. Environ. Pollut..

[61] Brewer G.J. (2010). Risks of copper and iron toxicity during aging in humans. Chem. Res. Toxicol..

[62] Taylor A.A., Tsuji J.S., Garry M.R., McArdle M.E., Goodfellow W.L., Adams W.J., Menzie C.A. (2020). Critical review of exposure and effects: Implications for setting regulatory health criteria for ingested copper. Environ. Manag..

